# Pulmonary tuberculosis presenting secondary organizing pneumonia with organized polypoid granulation tissue: case series and review of the literature

**DOI:** 10.1186/s12890-020-01292-7

**Published:** 2020-09-22

**Authors:** Eun Jin Kim, Kyung Chan Kim

**Affiliations:** Department of Internal Medicine, Daegu Catholic University Medical Center, Daegu Catholic University School of Medicine, 33, Duryugongwon-ro 17-gil, Nam-gu, Daegu, 42472 South Korea

**Keywords:** Tuberculosis, Organizing pneumonia, Transbronchial lung biopsy

## Abstract

**Background:**

Secondary organizing pneumonia (SOP) is difficult to distinguish from cryptogenic organizing pneumonia (COP) considering various clinical situations. SOP caused by *Mycobacterium tuberculosis* is rare; indeed, it has not been reported as a sequela of disseminated tuberculosis.

**Methods:**

From January 2016 to December 2018, we identified six cases of tuberculosis-associated SOP in which *Mycobacterium tuberculosis* was revealed by microbiological examination; one of the cases was miliary tuberculosis.

**Results:**

Of the six cases, 17% were positive for acid fast bacillus (AFB) stain, but 100% were positive for *M. tuberculosis* polymerase chain reaction (MTB PCR) and AFB culture. In all cases, transbronchial lung biopsy was performed and organizing pneumonia was confirmed pathologically. All survived after treatment with anti-tuberculosis therapy.

**Conclusions:**

Pulmonary tuberculosis, which shows OP in lung biopsy, is diagnosed through MTB PCR and AFB culture, and the prognosis is thought to be good.

## Background

Organizing pneumonia (OP) is pathologically defined as the presence of organized polypoid granulation tissue in alveolar ducts and alveoli bronchioles, with or without bronchiolar organization [1, 2]. OP is classified as cryptogenic (idiopathic) OP (COP) or secondary OP (SOP). SOP refers to OP caused by a known cause, and representative examples include infection, connective tissue disease, drugs, inflammatory bowel disease, hematologic malignancies, organ transplantations and radiation therapy [2, 3]. Post-respiratory infection SOP occurs after an infectious pneumonia, such as viruses, bacteria, fungi, or parasites [4, 5]. It can also be caused by *Mycobacterium tuberculosis*, although rarely [6–9]; to date, disseminated tuberculosis has not been reported as SOP. According to statistics in 2017, Korea is a country with a prevalence of tuberculosis in 70 people per 100,000 population, but so far there has been only one case report on tuberculosis-related OP. Here, we report six cases of SOP that occurred after *M. tuberculosis* infection, including disseminated tuberculosis, and review the current literature.

## Methods

This retrospective study is based on a review of medical records of patients that underwent lung biopsy at the Department of Respiratory Medicine at Daegu Catholic University Medical Center between January 2016 and December 2018 to confirm OP. In total, 85 OP cases were identified pathologically, and microbiological data obtained during the same period was collected to confirm SOP. SOP was 66 (77.6%) out of 85 cases, with infection-related SOP (54 cases) including pulmonary tuberculosis, followed by cancer, radiation therapy, connective tissue disease, and drugs. The study was approved by the local institutional review board (No. CR-19-069). The requirement for informed consent was waived.

Microbial information was confirmed by acid fast bacillus (AFB) smear, AFB culture, and *M. tuberculosis* polymerase chain reaction (MTB PCR) of sputum, bronchial wash fluid, and tissue (if necessary) to prove the presence of *M. tuberculosis*. Pulmonary tuberculosis was diagnosed by MTB PCR of bronchial wash fluid or sputum; false-positives were eliminated by cross referencing the PCR results with clinical, AFB smear, and culture results. In addition, even if the AFB smear was positive, samples were further tested using MTB PCR or AFB culture prior to a final diagnosis of pulmonary tuberculosis.

Lung biopsy was performed when the pneumonia was in the form of a mass, there was no improvement in treatment, or the pneumonia pattern needed to be differentiated from other diseases.

The lung biopsy results revealed six SOP cases with proven pulmonary tuberculosis. Patient characteristics, symptoms, smoking history, underlying disease, medication history, laboratory test results, chest radiographs, treatments, response to treatment, drug sensitivity test using absolute concentration method, and death were investigated retrospectively. All patients were followed up for 12 months. In addition, we reviewed all articles reporting SOP related to pulmonary tuberculosis.

## Results

**Fig. 1:**
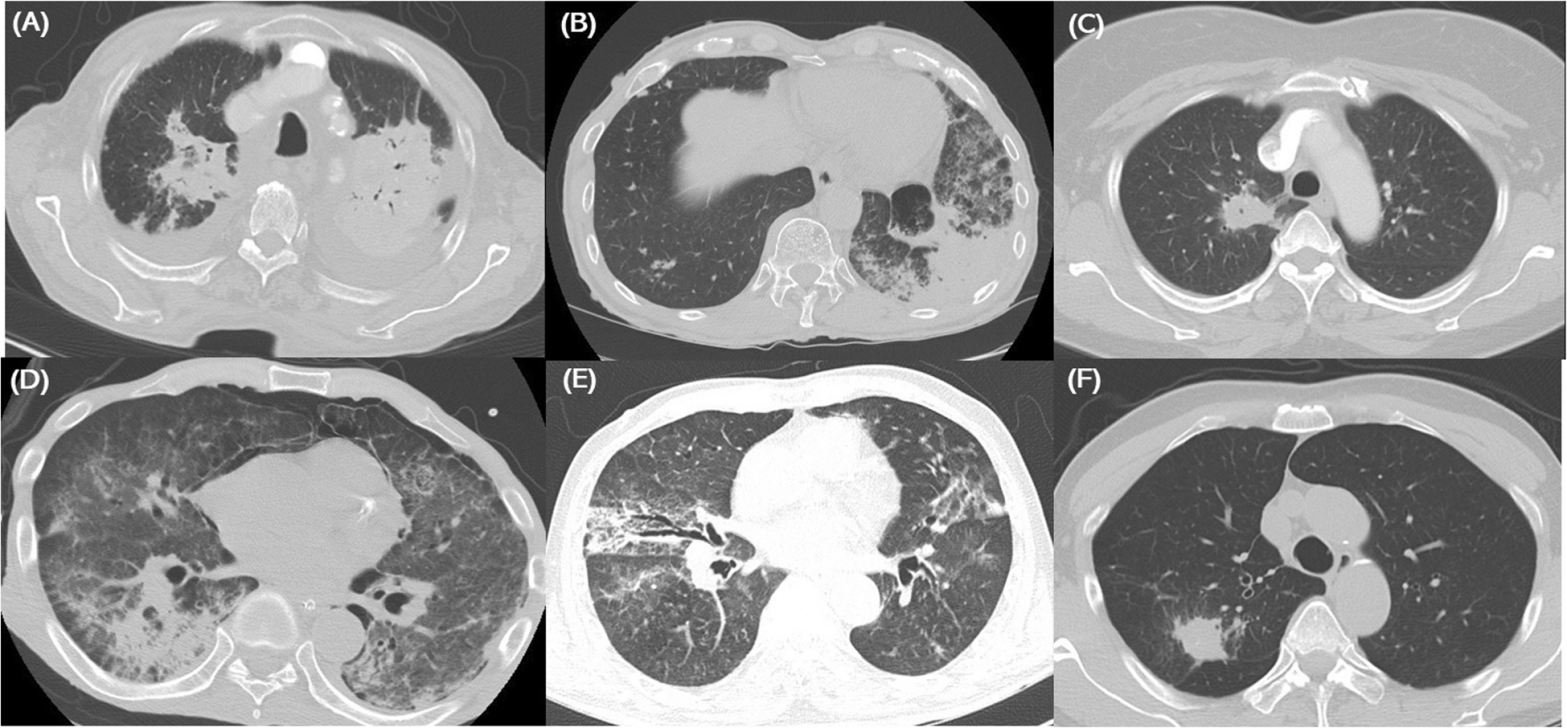
Chest computed tomography (CT) scan features of all six cases. **a** Case 1. Consolidation in both upper lobes and pleural effusion. Transbronchial lung biopsy (TBLB) was performed in the left upper lobe. **b** Case 2. Consolidation in the left lower lobe, which was subjected to TBLB. **c** Case 3. A thick-walled cavity is present in the right upper lobe, which was subjected to TBLB. **d** Case 4. Consolidation in the right lower and left lower lobes. TBLB was performed in the right lower lobe. **e** Case 5. Multiple nodules with patchy consolidation in both lungs. TBLB was performed in the right middle lobe. **f** Case 6. A solitary pulmonary nodule in the right upper lobe, which was subjected to TBLB

**Fig. 2:**
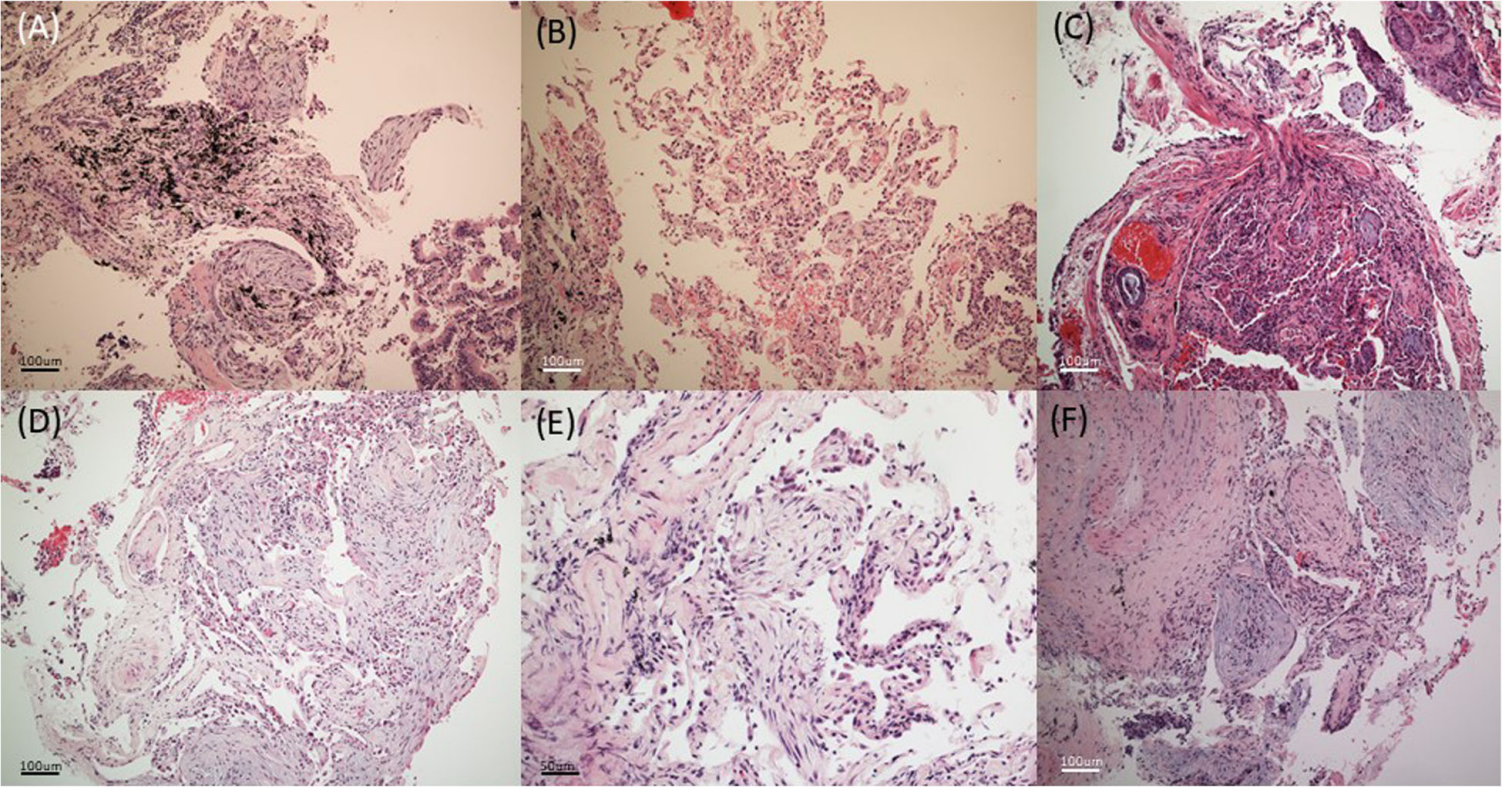
Lung pathologic features of all six cases. There were organizing polypoid granulation tissue plugs within alveolar ducts and terminal bronchioles. **a** Case 1, **b** Case 2, **c** Case 3, **d** Case 4, **f** Case 6. H & E stain, X 100. **e** Case 5. X200

**Fig. 3:**
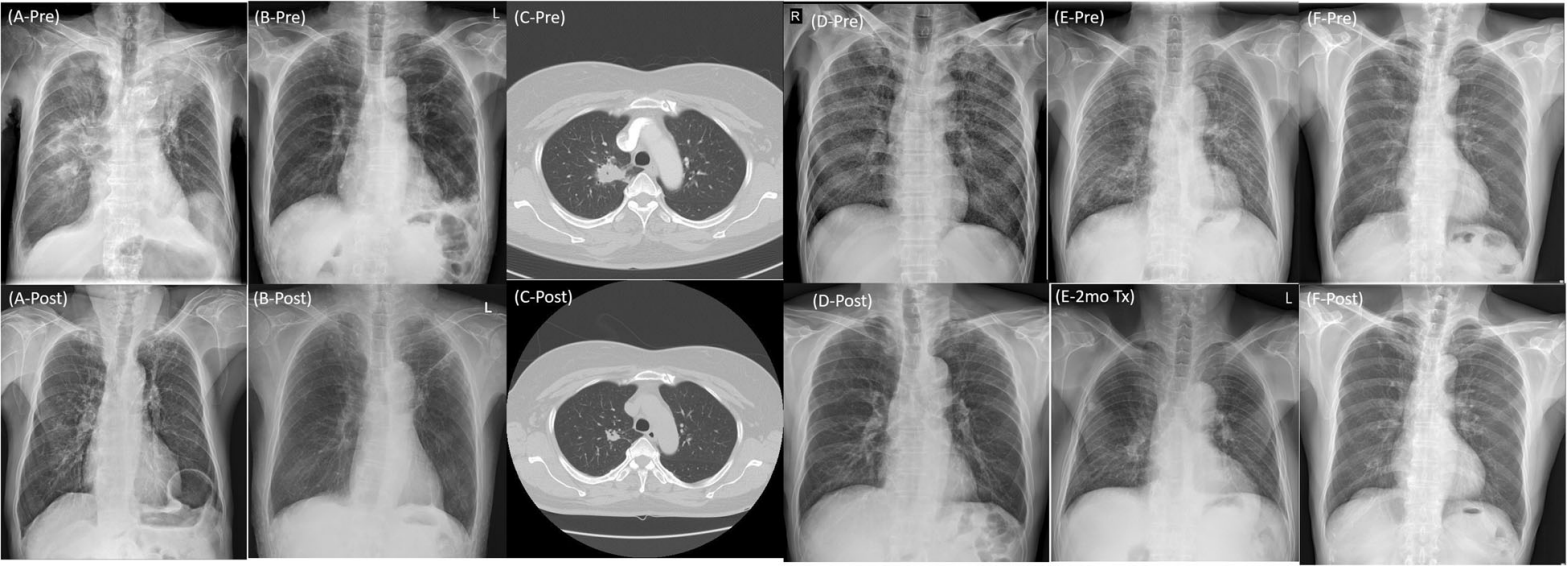
Pre-treatment and post-treatment chest images of all six cases. **a** Case 1, **b** Case 2, **c** Case 3, **d** Case 4, **e** Case 5, **f** Case 6. In each case, ‘-Pre’ means before treatment and ‘-Post’ means after treatment. ‘E-2mo Tx’ means chest X ray at 2mo of treatment in case 5

The case series are summarized in Table 1. The Fig. 1 shows the radiologic features, and the Fig. 2 shows the pathologic features of these cases. The Fig. 3 shows the chest X ray (CXR) and computed tomography (CT) before and after treatment.

**Table 1:** Characteristics of patients with organizing pneumonia associated with *Mycobacterium tuberculosis* infection

	Case 1	Case 2	Case 3	Case 4	Case 5	Case 6
Sex/age	M/81	M/71	F/53	M/57	M/78	M/70
Symptom	General weakness, Fever	Chronic cough	Fever	Dyspnea	Mental status change	Asymptomatic
Past history	Hypertension	Neurofibromatosis, GIST, old pulmonary tuberculosis	Diabetes, Hyperlipidemia	(−)	Spinal disease	(−)
Previous medication	Losartan	Cilostazol, choline alfoscerate, nebivolol, olmesartan, pantoprazole, udenafil	Metformin, sitagliptin, rosuvastatin, tibolone, valsartan, pioglitazone, glimepiride	(−)	Naproxen, eperisone, mecobalamin, gabapentin, limaprost, esomeprazole	(−)
Smoking	(−)	(−)	(+) 10pack-years	(+) 20pack-years	(−) Ex-smoker. 25pack-years	(−) Ex-smoker, 10pack-years
Chest CT	Bilateral consolidation	Consolidation in LLL	Consolidation in RUL	Bilateral consolidation	Bilateral consolidation	Nodule
Sputum						
AFB smear	(−)	(−)	(−)	(−)	(−)	(−)
Bronchial washing or BAL fluid						
AFB smear	(+)	(−)	(−)	(−)	(−)	(−)
MTB PCR	(+)	(+)	(+)	(+)	(+)	(+)
AFB Culture	(+)	(+)	(+)	(+)	(+)	(+)
Biopsy	OP with a few of small granuloma	OP	OP	OP	OP	OP
Tissue AFB smear	(−)	N/A	N/A	N/A	N/A	N/A
BAL	(−)	(+) cell count 550/μL, neutrophil 61%, lymphocyte 11%	(−)	(+) cell count 150/μL, neutrophil 3%, lymphocyte 45%	(+) cell count 450/μL, neutrophil 6%, lymphocyte 29%	(−)
Ventilator Tx	(−)	(−)	(−)	(+)	(−)	(−)
DST	All sensitive	All sensitive	All sensitive	All sensitive	All sensitive	All sensitive
Antituberculosis drugs	Isoniazid, rifampin, ethambutol, pyrazinamide, for 6 months	Isoniazid, rifampin, ethambutol, pyrazinamide for 6 months	Isoniazid, rifampin, ethambutol, pyrazinamide for 6 months	Isoniazid, rifampin, ethambutol, pyrazinamide ➔ isoniazid, pyrazinamide, levofloxacin for 18 months	Isoniazid, rifampin, ethambutol, pyrazinamide for 2 months ➔ quit the medicine by himself	Isoniazid, rifampin, ethambutol, pyrazinamide for 6 months
Treatment of steroid	(−)	(+)	(−)	(+)	(−)	(−)
Resolution	(+)	(+)	(+)	(+)	(+)	(+)
Recur	(−)	(−)	(−)	(−)	(−)	(−)
Mortality	Alive	Alive	Alive	Alive	Alive	Alive

*GIST* gastrointestinal stromal tumor, *BAL* bronchoalveolar lavage, *AFB* acid fast bacillus, *DST* Drug sensitivity test, *OP* organizing pneumonia, *N/A* not available, *LLL* left lower lobe, *RUL* right upper lobe

### Case 1

An 81-year-old male visited hospital with a 3-day history of anorexia, fever, cough, and phlegm. Examination at the time of the visit revealed the following: blood pressure (BP), 137/62 mmHg; pulse rate (PR), 103 beats/min; respiratory rate (RR), 20 breaths/min; body temperature (BT), 37.7 °C, and peripheral saturation, 95% on room air. CXR (Fig. 3a-Pre) and CT (Fig. 1a) revealed lobar consolidation in both upper lung fields, focal consolidation in right middle and right lower lobes, and bilateral pleural effusion. A complete blood count revealed the leukocyte number to be 7400/uL (neutrophils, 81.9%). Other laboratory values were as follows: hemoglobin (Hb), 9.8 g/dL; erythrocyte sedimentation rate (ESR), 20 mm/hr. (normal range, 0–10 mm/hr); C-reactive protein (CRP), 45.5 mg/L (normal, < 5 mg/L); procalcitonin, 0.14 ng/mL (normal < 0.5 ng/mL); pro-B-type natriuretic peptide (pro-BNP), 1271 pg/mL (normal range, 17.5–158.2 pg/mL); Na, 130 mEq/L; blood urea nitrogen (BUN), 5.9 mg/dL; creatinine (Cr), 0.7 mg/dL; and albumin, 1.6 g/dL. An anti-HIV antibody screening test was negative. After starting antibiotic treatment for community acquired pneumonia, a sputum AFB smear was negative, but the sputum MTB PCR was positive. Therefore, he received a diagnosis of pulmonary tuberculosis and was treated with isoniazid, rifampin, ethambutol, and pyrazinamide. After 2 weeks, CXR was unable to rule out malignancy and did not improve, so CT was taken. We performed transbronchial lung biopsy (TBLB) and bronchial washing under radial probe endobronchial ultrasonography (R-EBUS). The biopsy revealed OP with small granuloma (Fig. 2a). Anti-tuberculosis treatment was continued due to a positive AFB stain and MTB PCR of the bronchial wash fluid. Subsequent AFB culture confirmed *M. tuberculosis*, which was sensitive to all anti-tuberculosis drugs. No steroid was used. A further follow-up CXR (Fig. 3a-Post) showed an improvement in his condition.

### Case 2

A 72-year-old male visited the hospital with a recent history of cough, phlegm and increased focal opacity in of left lower lung field (LLLF) on CXR (Fig. 3b-Pre). On admission, BP was 120/70 mmHg, PR was 90/min, RR was 20/min, and BT was 36.6 °C. Chest CT showed consolidation in the LLLF (Fig. 1b). The leukocyte count was 4700/uL (neutrophils, 58%), Hb was 12.6 g/dL, ESR was 26 mm/hr., CRP was 5.3 mg/L, procalcitonin was 0.03 ng/mL, Na was 139 mEq/L, BUN was 15.1 mg/dL, Cr was 0.8 mg/dL, and albumin was 2.8 g/dL. An anti-HIV antibody screening test was negative. To confirm the cause of pneumonia, TBLB and bronchoalveolar lavage (BAL) were performed under R-EBUS. The lung biopsy results suggested OP (Fig. 2b). AFB stain of bronchial wash fluid was negative, but the MTB PCR was positive. Since the biopsy and radiologic examination results did not show findings appropriate for TB, the MTB PCR result was determined to be a false-positive. Therefore, he received corticosteroid treatment while maintaining antibiotic treatment. CXR showed that LLLF infiltration improved after steroids, but 3 weeks later *M. tuberculosis* was confirmed on AFB culture of BAL fluid. Therefore, the steroid was discontinued and anti-tuberculosis treatment was started. The microbe was sensitive to all anti-tuberculosis drugs and CXR revealed that all lesions improved thereafter (Fig. 3b-Post).

### Case 3

A 54-year-old female patient was transferred to the clinic due to identification of a speculated mass measuring 3.2 × 2.8 cm in right upper lobe (RUL) on CXR and chest CT (Fig. 1c), accompanied by a fever lasting 4 days. Upon hospitalization, BP was 120/70 mmHg, PR was 78/min, RR was 20 /min, and BT was 36.9 °C. The leukocyte count was 7500/uL (neutrophils, 51.9%), Hb was 15.3 g/dL, ESR was 6 mm/hr., CRP was 1.4 mg/L, Na was 142 mEq/L, BUN was 16 mg/dL, Cr was 0.6 mg/dL, and albumin was 4.7 g/dL. No anti-HIV antibody screening test was performed. Antibiotic treatment was based on a diagnosis of pneumonia, and TBLB and bronchial washing were performed under R-EBUS to exclude malignancy. The biopsy results revealed OP (Fig. 2c). AFB stain of the bronchial wash fluid was negative, but MTB PCR was positive. Therefore, we made a diagnosis of pulmonary tuberculosis and began anti-tuberculosis treatment. An AFB culture test confirmed *M. tuberculosis*, which was sensitive to all drugs. No steroids were used. After 6 months of anti-tuberculosis treatment, chest CT revealed that the mass lesions on the RUL had improved (Fig. 3c-Post).

### Case 4

A 57-year-old male patient visited the emergency room with a 3-week history of generalized weakness and dyspnea. At the time of the visit his BP was 134/72 mmHg, his PR was 118/min, his RR was 20/min, his BT was 36.1 °C, and room air oxygen saturation was 90%. CXR (Fig. 3d-Pre) and Chest CT revealed multiple micronodules distributed randomly in both lungs, accompanied by cavitary nodules, irregular linear opacity, and patchy consolidation in both apices. He was diagnosed with miliary tuberculosis with active pulmonary tuberculosis. The leukocyte count was 4500/uL (neutrophils, 93.2%), Hb was 14.5 g/dL, ESR was 8 mm/hr., CRP was 213 mg/L, procalcitonin was 17.65 ng/mL, Na was 114 mEq/L, BUN was 81.2 mg/dL, Cr was 2 mg/dL, and albumin was 2.9 g/dL. An anti-HIV antibody screening test was negative. Sputum AFB stain was negative, but MTB PCR was positive; therefore, he received anti-tuberculosis drugs (isoniazid, rifampin, ethambutol and pyrazinamide). Follow-up chest CT revealed increased diffuse consolidation and ground glass opacity (GGO) in both lungs (Fig. 1d). Mechanical ventilator treatment began on the 22nd day of hospitalization due to hypoxia. To confirm the cause of the exacerbation we performed a blind TBLB and BAL. The BAL AFB stain was negative, and the MTB PCR results were positive. The biopsy results confirmed OP (Fig. 2d) and so he received corticosteroids while continuing anti-tuberculosis treatment. Subsequently, as hypoxia and CXR improved, the patient was extubated on the 12th day of ventilator treatment. *M. tuberculosis* was cultured in sputum and was confirmed to be sensitive to all drugs. The steroid was tapered for a total of 6 months and although anti-tuberculosis drug treatment continued, rifampin was stopped due to leukopenia and ethambutol was stopped due to impaired vision. The patient received anti-tuberculosis treatment (isoniazid, pyrazinamide, and levofloxacin) for 18 months. After treatment was completed, CXR showed that all lesions had improved (Fig. 3d-Post).

### Case 5

A 78-year-old male was admitted to the emergency room due to fainting and near drowning while sitting in a bathtub. At the time of the visit, his BP was 100/60 mmHg, his PR was 126/min, his RR was 23/min, his BT was 37.8 °C, and his peripheral saturation was 83% on nasal O2 (4 L/min). Chest CT revealed suspected pulmonary edema with diffuse patchy consolidations and GGO with a crazy paving pattern in both lungs. The leukocyte count was 5400/uL (neutrophils, 61.3%), Hb was 11.3 g/dL, ESR was 6 mm/hr., CRP was 0.6 mg/L, procalcitonin was 13.16 ng/mL, BNP was 253 pg/mL, Na was 131 mEq/L, BUN was 13.1 mg/dL, and Cr was 0.9 mg/dL. The anti-HIV antibody screening test was negative. The patient’s medical history and imaging findings suggested a high probability of pulmonary edema, but procalcitonin was high and a mild fever of > 37.8 °C persisted. Therefore, antibiotic treatment was started for suspected pneumonia, along with diuretics. CXR (Fig. 3e-Pre) showed new infiltration in both lungs, so Chest CT (Fig. 1e) was followed up on the 10th day of hospitalization. In chest CT, new diffuse centrilobular nodules with multifocal conglomerated nodules were observed in both lungs. TBLB and BAL were performed under R-EBUS to identify the cause. AFB stain of the BAL fluid was negative, but the MTB PCR was positive; therefore, anti-tuberculosis drugs were started. The biopsy confirmed OP (Fig. 2e), but no steroids were given. *M. tuberculosis* was identified on BAL AFB culture, which was sensitive to all drugs. Anti-tuberculosis drugs were used for 2 months. CXR tracking confirmed that the lesions improved (Fig. 3e-2mo Tx). However, side effects occurred and, despite providing medical care about side effects, he stopped all medications on his own after 2 months. The patient is just being followed as he wanted.

### Case 6

A 70-year-old male visited the clinic due to identification of a 2.7 cm solitary pulmonary nodule on CXR (Fig. 3f-Pre) and CT (Fig. 1f). At the time of the visit, his BP was 121/74 mmHg, his PR was 62/min, his RR was 20/min, and his BT was 36.4 °C. The leukocyte count was 6200/uL (neutrophils, 57.8%), Hb was 12.1 g/dL, Na was 134 mEq/L, BUN was 9.7 mg/dL, Cr was 0.8 mg/dL, and albumin was 4.4 g/dL. No anti-HIV antibody screening test was performed. No antibiotics were used. TBLB and bronchial washing were performed under R-EBUS; the biopsy confirmed OP (Fig. 2e). AFB staining of the bronchial washing fluid was negative, but the MTB PCR was positive, so anti-tuberculosis treatment was started due to a diagnosis of pulmonary tuberculosis. *M. tuberculosis* was confirmed by AFB culture and was sensitive to all drugs. Steroids were not used. After 6 months of anti-tuberculosis treatment, CXR revealed that the RUL nodular lesion had improved (Fig. 3f-Post).

## Discussion

OP is triggered by lung injury. The alveolar epithelium reacts to lung damage and produces granulation tissue. Inflammatory debris fills the alveoli, alveolar ducts, and terminal bronchioles, with characteristic endoluminal buds of granulation tissue known as the Masson body [10]. SOP is caused by infection-induced lung injury, drug toxicity, inhalation of a pathogen (e.g., cocaine), inhalation of toxic gas, gastroesophageal reflux, connective tissue disorders, organ transplantation, or radiation [3, 11, 12], but SOP caused by tuberculosis is rare. Here, we describe six cases (Table 1) of tuberculosis-related SOP and conducted a literature review (Table 2).

**Table 2:** Literature review of cases of organizing pneumonia associated with *Mycobacterium tuberculosis* infection

	Lawn et al. [5]	Sander et al. [6]	Yoon et al. [7]		Hsieh et al. [8]
No. of cases	One case	One case	Two cases		One case (only image)
Sex/age	F/27	M/75	F/78	F/75	M/80
Past medical history	AIDS	Hypertension, atrial fibrillation, colon polyp.	–	–	N/A
Bronchial wash or sputum					
AFB smear	–	–	–	+	–
MTB PCR	N/A	N/A	+	–	N/A
AFB culture	+	+	+	+	+
DST	N/A	N/A	All sensitive	Isoniazid resistant	Isoniazid resistant
Biopsy	OP with AFB smear (+)*	OP	OP	OP	OP with AFB smear (+)
Steroid treatment	Not used	Used before and after diagnosis of tuberculosis	Not used	Not used	N/A
Prognosis	Dead due to fatal ARDS before diagnosis and treatment	Improved	Improved	Improved	N/A

*AFB* acid fast bacillus, *DST* drug sensitivity test, *OP* organizing pneumonia, *N/A* not available, *ARDS* acute respiratory distress syndrome

*Postmortem biopsy

### Symptoms and microbial test results

COP has an acute and subacute course. Symptoms include coughing, dyspnea, and fever, meaning that it can easily be mistaken for infectious pneumonia and treated initially with antibiotics for several weeks [13, 14]. However, even if OP is diagnosed by performing a biopsy after a patient exhibits slow clinical improvement, efforts should be made to differentiate the cause, bearing in mind the possibility of SOP. Cases 1–3 and case 5 reported herein were treated initially with antibiotics due to suspected pneumonia. Although the biopsy results of all four patients confirmed OP, and all AFB stains of the initial respiratory samples were negative, it was possible to initiate anti-tuberculosis treatment relatively early because the MTB PCR was positive [15]. It should be borne in mind that tuberculosis is a possible cause of OP in regions such as Korea, which have a relatively high prevalence of tuberculosis. Therefore, when performing a bronchoscopy to obtain a biopsy, a tuberculosis test such as MTB PCR will help to confirm whether SOP is present.

Our literature review to collect articles reporting OP associated with *M. tuberculosis* infection identified five cases published worldwide (Table 2) [6–9]. The AFB smear of bronchial washing fluid was positive in only one case, reported by Yoon et al. [8]; it was negative in bronchial washing fluid or sputum in the other four cases [6–9]. MTB PCR of bronchial washing fluid was positive in one case because MTB PCR was performed in only two cases [8]. All lung tissue had OP histologically, but in only two cases was the AFB smear positive in lung tissue [6, 9]. Eventually, all cases were AFB culture-positive in bronchial washing fluid or sputum, confirming a diagnosis of tuberculosis.

Taking the six cases reported herein along with the other five reported worldwide, only 18% showed a positive result (i.e., OP associated with *M. tuberculosis* infection) for the AFB smear of respiratory samples. MTB PCR of respiratory specimens was positive in seven out of eight cases (87.5%). AFB culture was positive for all respiratory specimens. In areas in which the prevalence of tuberculosis is high, confirming MTB PCR with a respiratory sample is likely to make the diagnosis faster even if the biopsy revealed OP.

### Biopsy methods

In our study, case 4 underwent blind TBLB because the lesion was diffuse; the other five cases had localized lesions and, after confirming the lesion with R-EBUS without fluoroscopy, TBLB was performed. It was possible to obtain an appropriate sample without pneumothorax by performing R-EBUS [16]. Ma et al. [17] reported that R-EBUS increases the accuracy of OP diagnosis, with fewer side effects. OP is best diagnosed from a surgical lung biopsy, but this is associated with an increased risk of morbidity and mortality. Many studies reported improvements in treatment decision making after diagnosis of OP with TBLB [4, 18, 19]. OP is more likely to appear as GGO or consolidation on the chest CT than as a nodule or mass [10, 20]. Often, tissue biopsy via bronchoscopy is often performed blind; in such cases it is difficult to obtain tissue from an accurate location. In our study, an exact histological diagnosis of OP was possible in the five patients who underwent R-EBUS-guided TBLB due to accurate localization of the lesion.

### Treatments

In our study, all six cases received anti-tuberculosis treatment, which improved the lung lesions. However, in case 2, the lung lesions improved after administration of steroids prior to anti-tuberculosis treatment. In case 4, the condition progressed to acute respiratory distress syndrome (ARDS) during anti-tuberculosis treatment; the cause was confirmed to be OP, so pulmonary lesions improved after administration of steroids together with anti-tuberculosis treatment. Among the articles identified in the literature review (Table 2), we found two cases that improved after anti-tuberculosis treatment without steroid treatment [8]. However, there was one case in which the pulmonary lesion was improved after administration of steroids before tuberculosis was diagnosed [7]; this is similar to case 2 in our study. Lawn et al. [6] reported a case of ARDS similar to case 4 in our study; OP and tuberculosis were demonstrated at postmortem biopsy. In this case, steroid use may have helped improve the prognosis. In case 4, despite the use of anti-tuberculosis drugs after a diagnosis of military tuberculosis, ARDS developed and the improved after steroid administration.

The occurrence of pathological reactions in tuberculosis is inextricably linked with the host’s response to the invading *M. tuberculosis*. The immunologic response to *M. tuberculosis* is likely responsible for the characteristic presentation of tuberculosis [21]. As in case 4, in the case of a patient with insufficient Immune response, it would have been expressed in the form of a disseminated infection, although not human immunodeficiency virus (HIV). In this case, it is thought that the pulmonary inflammation worsened after using tuberculosis drugs, such as immune reconstitution inflammatory syndrome (IRIS).

The use of corticosteroids in pulmonary tuberculosis has been and remains controversial. A controlled trial reported by Johnson et al. [22] demonstrated that corticosteroid treatment most benefited the seriously ill patient (defined by low serum albumin concentration, low body weight, and severe weight loss) who had extensive tuberculosis. This benefit was evidenced mainly by an increase in the rate of radiographic clearing; there was no adverse effect on the bacteriologic response. Similar results can be found in a systemic review [23]. And steroids may also be of benefit in patients with marked abnormalities of gas exchange and respiratory failure [24, 25]. But, in less severely ill patients, methylprednisolone was either of no benefit or actually decreased the speed of sputum conversion [25]. The fact that steroids can be helpful when ARDS worsen despite anti-tuberculosis treatment is probably due to the lung pathology as OP.

All cases in our study were sensitive to antibiotics used to treat tuberculosis and all survived. Therefore, tuberculosis-associated SOP has a good prognosis.

All the patients were told about their disease and received anti-tuberculosis treatment (isoniazid, rifampin, ethambutol, pyrazinamide). However, a case 5 patient refused to treat anti-tuberculosis medication due to his side effects (neuralgia and anorexia), which did not finish the entire drug duration despite the side effects treatment.

### Biopsy features

If AFBs is transported to and reached the lungs, but the host’s defense system fails to clear the infection, the AFB proliferates inside the alveolar macrophages and kills the cells. Infected macrophages produce cytokines that attract other phagocytes, eventually forming nodular granulomas. Histologically, tuberculosis is characterized by granuloma, which is a cellular aggregate; macrophages and lymphocytes are important for formation of granulomas [26, 27]. Granulomas have been traditionally considered to be host-protective structures, thought to “wall off” bacteria and keep them from disseminating. Whereas this view may apply in later stages of fibrotic and calcified granulomas, early granuloma formation actually promotes infection by facilitating cell-to-cell spread within the macrophage aggregates, thus optimizing expansion of the bacterial population [25]. However, OP is characterized histologically by fibrosis and organized granulation tissue in the alveoli; fibroblasts play an important role and are recruited in response to alveolar epithelial cell injury [4]. In case 1, OP and granuloma were present; according to Huo et al. [28], the presence of epithelioid cell granulomas or giant multinucleated cells in OP is associated with an infectious etiology [7]. In this case, it is predicted that, in some areas, an immune response to typical tuberculosis occurs and, in some areas, fibroblasts are activated due to alveolar epithelial cell injury, thus showing up as OP. In cases 2–6, we could not confirm granuloma histologically; since we cannot biopsy all the lungs, we believe that the area with OP is biopsied. Granulomatous formation caused by a cellular immune response may be in the lungs that are not biopsied. Further research is needed.

Since this case series showed small numbers of cases, it may be limited in general application.

## Conclusions

Pulmonary tuberculosis can be a cause of SOP. In areas with high tuberculosis prevalence, we suggest that performing tuberculosis-related tests, especially MTB PCR, is useful for early diagnosis and treatment of tuberculosis related OP, even if the lung biopsy results show OP. The prognosis of tuberculosis related OP is thought to be good.

## Data Availability

All authors had access to data and material and vouch for its complete accuracy. Literature review can be accessed through Pubmed and Google Scholar. Data and materials can be accessed through the medical records at Daegu Catholic University Medical Center. All images are available through PACS imaging system storage at Daegu Catholic University Medical Center.

## Abbreviations

OP: Organizing pneumonia
COP: Cryptogenic organizing pneumonia
SOP: Secondary organizing pneumonia
AFB: Acid fast bacillus
MTB PCR: *Mycobacterium tuberculosis* polymerase chain reaction
BP: Blood pressure
PR: Pulse rate
RR: Respiration rate
BT: Body temperature
CXR: Chest X ray
CT: Computed tomography
Hb: Hemoglobin
ESR: Erythrocyte sedimentation rate
CRP: C-reactive protein
Pro-BNP: Pro-B-type natriuretic peptide
Na: Sodium
BUN: Blood urea nitrogen
Cr: Creatinine
TBLB: Transbronchial lung biopsy
R-EBUS: Radial probe endobronchial ultrasound
LLLF: Left lower lung field
BAL: Bronchoalveolar lavage
RUL: Right upper lobe
