# Correction to: Pulmonary tuberculosis presenting secondary organizing pneumonia with organized polypoid granulation tissue: case series and review of the literature

**DOI:** 10.1186/s12890-022-01938-8

**Published:** 2022-04-28

**Authors:** Eun Jin Kim, Kyung Chan Kim

**Affiliations:** Department of Internal Medicine, Daegu Catholic University Medical Center, Daegu Catholic University School of Medicine, 33, Duryugongwon-ro 17-gil, Nam-gu, Daegu, 42472 South Korea

## Correction to: BMC Pulmonary Medicine (2020) 20:252 https://doi.org/10.1186/s12890-020-01292-7

Following publication of the original article [1], the authors flagged that the following funding statement had been omitted from the Funding declaration: This work was supported by research grants from Daegu Catholic University in 2019.

The published article has now been updated with this declaration.
